# Primary carcinoid tumor arising within mature teratoma of the kidney: report of a rare entity and review of the literature

**DOI:** 10.1186/1746-1596-2-15

**Published:** 2007-05-17

**Authors:** Henry B Armah, Anil V Parwani

**Affiliations:** 1 Department of Pathology, University of Pittsburgh Medical Center, Pittsburgh, PA, USA

## Abstract

**Background:**

Primary carcinoid tumor arising within mature teratoma of the kidney is extremely rare, and their clinicopathologic features are not well described. Our objective was to further define the clinical features and pathologic spectra of primary carcinoid tumor arising within mature teratoma of the kidney.

**Methods:**

Six previously reported case reports were identified using MEDLINE and a subsequent bibliographic search of all pertinent reports and reviews was performed. We also searched the electronic medical archival records of our institution and identified one additional unreported case. Data were extracted on the demographics, predisposing factors, clinical presentation, radiographic features, gross pathology, microscopic pathology, immunophenotype, therapy, and outcome of each of these seven cases.

**Results:**

Primary carcinoid tumor arising within mature teratoma of the kidney was found at a mean age of 41.4 years. Of the 7 cases, 3 were female and 4 were male. Two of the 7 cases (28.6%) were associated with horseshoe kidney. It typically presented with abdominal pain without carcinoid syndrome. It typical radiologic appearance was well circumscribed partly calcified Bosniak II-III lesion. Histologically, the carcinoid tumor showed monotonous small round cells arranged in classic anastomosing cords/ribbons intermixed with solid nests. Surgery was curative, no additional treatment was required, no local recurrences occurred, and no metastases occurred in all 7 cases. The 3 cases with available outcome data were alive at the time of publication of their respective cases (mean, 5 months).

**Conclusion:**

Primary carcinoid tumor arising within mature teratoma of the kidney is a rare tumor that typically presents with abdominal pain without carcinoid syndrome. It is not associated with local recurrence and metastasis, is surgically curable, and has excellent prognosis.

## Background

Primary carcinoid tumor and mature teratoma involving the kidneys are rare. The two entities existing simultaneously in the same kidney are exceptionally unique and extremely rare. Only 6 cases of primary carcinoid tumor arising in a mature teratoma of the kidney have been reported in the world medical literature to date [1-6], since the association was first described in 1976 by Kojiro et al [2]. Although carcinoid tumors are found most commonly in the gastrointestinal tract, they are also found in the respiratory, hepatobiliary, and genitourinary systems. Approximately 60 cases of primary renal carcinoid tumor have appeared in the international medical literature [7,8], and 10 of these cases occurred in a horseshoe kidney [7]. Primary carcinoid tumor arising in a mature teratoma of the kidney is over-represented in patients with horseshoe kidney and other congenital developmental renal defects [1,3]. The clinical and pathologic features of primary carcinoid tumor arising in a mature teratoma of the kidney are not well described, and with the rarity of these cases, a diagnosis may be extremely difficult to render, particularly on limited biopsy material. Additionally, little information exists about its histogenesis, natural history, biologic behavior and prognosis due to its rarity. In this article we review the clinical and pathologic features of primary carcinoid tumor arising in a mature teratoma of the kidney, focusing on the histologic spectrum of these tumors. We include a case retrieved from the electronic medical archival records of our institution (described in the tables as "current case") that has not been published previously.

## Methods

Six prior case reports of primary carcinoid tumors arising within mature teratoma of the kidney were found in a MEDLINE search. Additionally, the computerized medical archival records of the University of Pittsburgh Medical Center (1986 to present) was searched and 1 additional unreported case of primary carcinoid tumor arising within mature teratoma of the kidney was found. Formalin-fixed, paraffin-embedded tissue sections and the following immunohistochemical stains from this case were reviewed: Synaptophysin (1:100, DakoCytomation, Carpinteria, California), CD 56 (1:200, Dako), CK 7 (1:200, Dako), CK 20 (1:200, Dako), Pancytokeratin cocktail (AE1/AE3, 1:500, Dako; CAM 5.2, 1:50, Becton Dickinson; MNF116, 1:50, Dako; and UCD/PR-10.11, 1:25, Zymed, San Francisco, California), Calretinin (1:100, Becton Dickinson, San Jose, California), Thyroxine (1:500, Dako), Chromogranin (1:200, Signet Pathology Systems, Inc, Dedham, Mass), Thyroid transcription factor (TTF)-1 (1:250, Ventana, San Francisco, California), S-100 protein (1:200, Ventana), Melan-A (1:500, Ventana), Insulin (1:200, Ventana), Estrogen receptor (ER) (1:250, Ventana), and Progesterone receptor (PR) (1:250, Ventana). Tissue sections that had been shown to be positive or negative for each marker were used as controls.

## Results

### Clinical features

Clinical characteristics of the 7 cases of primary carcinoid tumor arising within mature teratoma of the kidney are summarized in Table 1[1-6]. Primary carcinoid tumor arising within mature teratoma of the kidney occurred in both the young and the old (range, 23 to 65 years; mean age, 41.4 years), but the majority of cases (85.7%, 6/7) presented in the fourth to seventh decades. Of the 7 cases, there was no gender predilection with 3 being male and 4 being female. Two of the 7 cases (28.6%) were associated with horseshoe kidney. Four of the 7 cases (57.1%) of primary carcinoid tumor arising within mature teratoma of the kidney presented with abdominal or flank pain, 1 (14.3%) presented with fever, and 2 (28.6%) were asymptomatic. None of the 7 cases presented with signs and symptoms of carcinoid syndrome. Five of the 7 cases (71.4%) evaluated by computerized tomography (CT) revealed well-circumscribed partly calcified Bosniak II-III lesions with minimal contrast enhancement.

**Table 1:** Clinical characteristics of primary carcinoid tumor arising within mature teratoma of the kidney

**Source, Year**	**Age, year**	**Sex**	**Clinical Presentation**	Radiographic Features of Renal Mass*	**Association with Horseshoe Kidney**
Kojiro et al, 1976 [2]	40	Male	Epigastric pain, nausea, no carcinoid syndrome	IV&RP: Marked dilatation of left renal pelvis	No
Fetissof et al, 1984 [3]	65	Male	Fever, no carcinoid syndrome	IVU: Displaced left kidney and non-visualized right kidney	Yes
Lodding et al, 1997 [1]	23	Male	Abdominal pain, no carcinoid syndrome	CT: Calcification in horseshoe kidney	Yes
Yoo et al, 2002 [4]	30	Female	Abdominal pain, no carcinoid syndrome	CT: Dense calcification with minimal contrast enhancement	No
Kim et al, 2004 [5]	39	Female	Asymptomatic, incidental renal mass, no carcinoid syndrome	CT: Relatively well demarcated and incompletely marginated by a thin hypodense rim with globular calcifications	No
Kurzer et al, 2005 [6]	58	Female	Asymptomatic, incidental renal mass, no carcinoid syndrome	CT: Round, smooth, and well marginated, with two solid clumps of calcifications (Bosniak III lesion)	No
Current case	35	Female	Right flank pain, right costovertebral angle tenderness, no carcinoid syndrome	CT: Exophytic, round, well-circumscribed mildly complex hypodense with globular calcifications (Bosniak II lesion) RU&KUB: Incomplete filling of upper pole infundibulum	No

*IV&RP, intravenous and retrograde pyelograms; IVU, intravenous urogram; CT, computerized tomography; RU&KUB, retrograde urogram and kidney-ureter-bladder plain x-ray

### Pathologic features

Gross, microscopic, and immunohistochemical features of the 7 cases of primary carcinoid tumor arising within mature teratoma of the kidney are summarized in Table 2[1-6].

**Table 2:** Pathologic characteristics of primary carcinoid tumor arising within mature teratoma of the kidney

**Source, year**	**Macroscopic findings**		**Components of Mature Teratoma**	**Architectural patterns of carcinoid tumor**	**Cytologic features of carcinoid tumor**	Mitotic rate of carcinoid tumor*	Immuno-phenotype of carcinoid tumor*
						
	**Side/Gross**	**Site/Size, cm**					
Kojiro et al, 1976 [2]	Left/Polycystic with smooth and nodular external surface	NP/17	Mucous secretory glands, columnar epithelium, mature hyaline cartilage, smooth muscle	NP	Finely granular eosinophilic cytoplasm, uniform rounded nuclei with coarse chromatin and small nucleoli	0/10 HPF	NP
Fetissof et al, 1984 [3]	Right/Dilated sac filled with purulent exudate in horseshoe kidney with lower poles connected by broad isthmus	NP/2	Transitional and mucinous columnar epithelium with occasional cilia, smooth muscle, ossified chondroid plaques, nerve bundles with ganglion cells	Anastomosing ribbons intermixed with solid nests	NP	NP	SER^+^, GLU^+^, SOM^+^, ACTH^-^, CAL^-^, GAS^-^, INS^-^, MOT^-^, NEU^-^
Lodding et al, 1997 [1]	Right/Gray-white firm nodule in horseshoe kidney with broad connecting bridge in midline	Lower pole/2	Mature bone	Organoid	Uniform cells	NP	PCK^+^, NSE^+^, CHR^+^, SYN^+^, S-100^+^, SER^+^, PSA^-^
Yoo et al, 2002 [4]	Left/Well circumscribed, tan-pink, glistening, focally hemorrhagic, primarily solid	Lower pole/3.5	Mucinous tall columnar epithelium, mature smooth muscle, mature bone	Anastomosing ribbons intermixed with solid nests	Monotonous small cells with finely stippled chromatin and inconspicuous nucleoli	0/10 HPF	PCK^+^, NSE^+^, CHR^+^
Kim et al, 2004 [5]	Right/Well defined solid, yellowish tan	Upper and mid poles/3.5	Mucinous columnar and pseudostratified columnar epithelium with occasional cilia, mature bone	Trabecular and anastomosing ribbons or nests	NP	NP	PCK^+^, NSE^+^, CHR^+^, SYN^+^
Kurzer et al, 2005 [6]	Right/Cyst filled with tan-brown friable material and three irregular tan-brown stones	Mid-lower pole/1	Transitional, colonic, squamous, and non-specific cuboidal epithelium, mature adipose, focal osseous metaplasia	Nests and cords	Small cells with peripheral palisading	NP	CHR^+^
Current case	Right/Cyst with irregular pale tan wall and focal calcifications	Middle pole/2	Urothelial-type and colonic epithelium, focal mature bone	Trabecular and anastomosing ribbon-like nests	Monotonous small round cells with peripheral palisading, and "salt-and-pepper" chromatin pattern	0/10 HPF	SYN^+^, CD 56^+^, PCK^+^, THY^+^, CK 7^-^, CK 20^-^, CAR^-^, PR^-^, ER^-^, S-100^-^, Melan-A^-^, INS^-^, TTF-1^-^, CHR^-^,

*NP, not provided; HPF; high power field; SYN, synaptophysin; PCK, pancytokeratin; THY, thyroxine; CK, cytokeratin; CAR, calretinin; PR, progesterone receptor; ER, estrogen receptor; INS, insulin; TTF, thyroid transcription factor; CHR, chromogranin; NSE, neuron specific enolase; SER, serotonin; PSA, prostate specific antigen; GLU, glucagon; SOM, somatostatin; ACTH, adrenocorticotropic hormone; CAL, calcitonin; GAS, gastrin; MOT, motilin; NEU, neurotensin

### Gross findings

**Figure 1:**
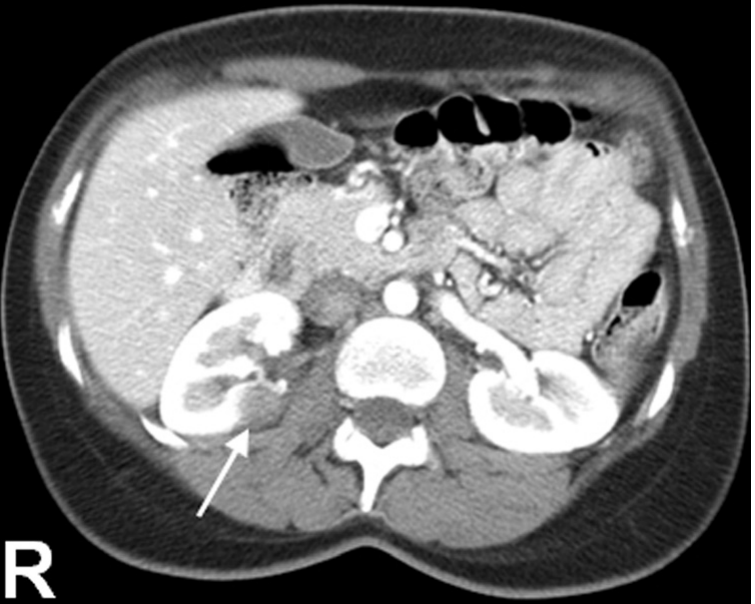
Contrast computed tomography scan of abdomen demonstrating right renal mass with calcifications (arrow). R indicates right side.

The size of the tumors in the 7 cases of primary carcinoid tumor arising within mature teratoma of the kidney ranged from 1 to 17 cm in greatest dimension (mean, 4.4 cm). Of the 7 cases, there was a right-sided predominance with 5 tumors involving the right kidney and 2 tumors involving the left kidney. The tumors were most commonly found in the middle and lower poles of the kidney. On gross examination, most of the tumors revealed circumscribed complex solid and cystic lesions with areas of calcification (Figure 1).

### Microscopic findings

**Figure 2:**
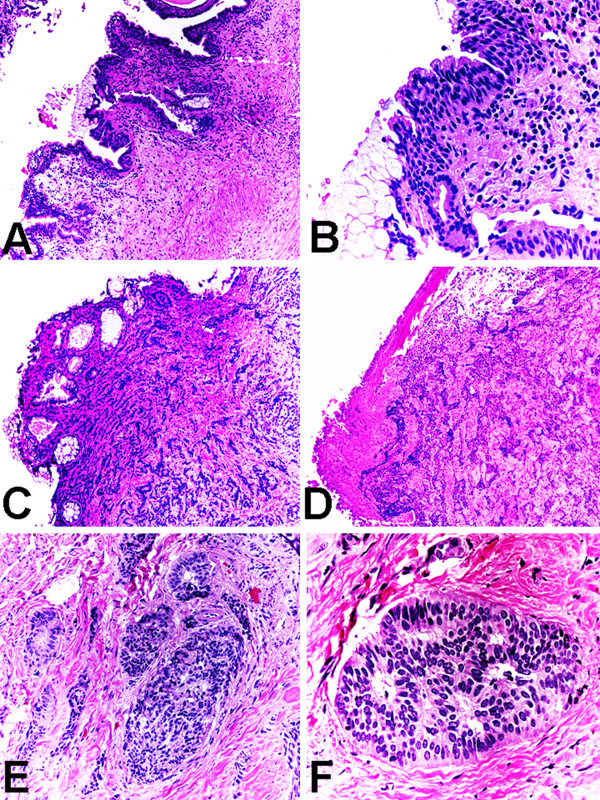
Histologic (H&E) findings of primary carcinoid tumor arising within mature teratoma of the kidney. (A) Teratomatous components of urothelium and mucinous columnar epithelium. Original magnification ×100. (B) Teratomatous components of urothelium and mucinous columnar epithelium. Original magnification ×400. (C) Carcinoid tumor underneath and closely apposed to the epithelial lining of the teratomatous cysts. Original magnification ×100. (D) Carcinoid tumor invading teratomatous mature bone. Original magnification ×100. (E) Carcinoid tumor showing the classical architectural pattern of trabecular and anastomosing ribbon-like nests. Original magnification ×200. (F) Carcinoid tumor showing the classic cytologic features of monotonous small cells with peripheral palisading and focal rosette-like arrangement, and "salt-and-pepper" chromatin pattern. Original magnification ×400.

Six of the 7 cases of primary carcinoid tumor arising within mature teratoma of the kidney showed the classic architectural pattern of trabecular and anastomosing ribbon-like nests (Figures 2C &2D), identical to the features of carcinoid tumors of other sites. Five of the 7 tumors showed the classic cytologic features of monotonous small cells (Figure 2E). The carcinoid tumor in our current case revealed proliferation of trabecular and anastomosing ribbon-like nests (Figure 2E), and the constituent tumor cells were monotonous small round cells with peripheral palisading and focal rosette-like arrangement, and "salt-and-pepper" chromatin pattern (Figure 2F). The mitotic rate of the tumor was quantified in 3 of the 7 cases as 0/10 high-power fields. The commonest teratomatous element in these 7 cases was mucinous columnar or colonic-like epithelium (Figures 2A, 2B &2C) in 6 cases, followed by mature bone (Figure 2D) in 4 cases, then smooth muscle in 3 cases, urothelium or urothelial-like epithelium (Figures 2A &2B) in 2 cases, cartilaginous tissue in 2 cases, nervous tissue in 1 case, and mature adipose tissue in 1 case. The carcinoid tumor in our current cases was underneath and closely apposed to the epithelial lining of the teratomatous cysts (Figure 2C) and invading teratomatous mature bone (Figure 2D).

### Immunohistochemical findings

**Figure 3:**
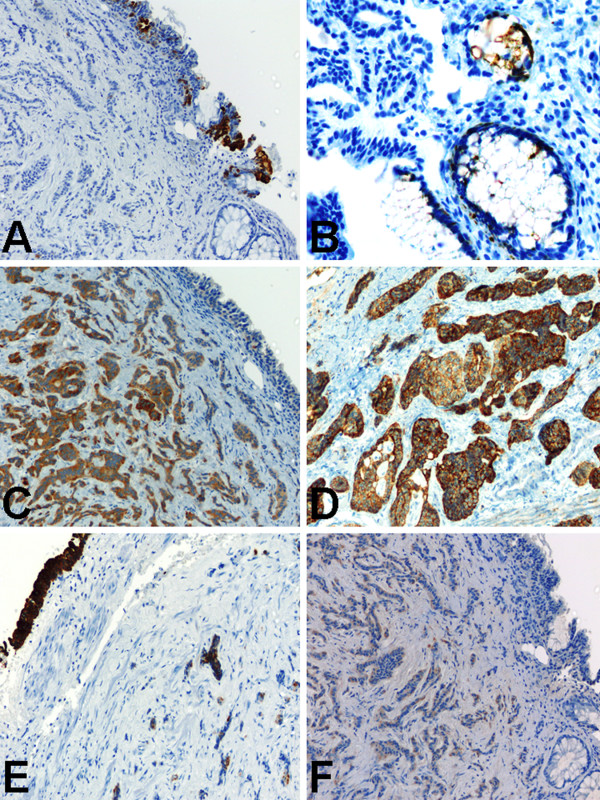
Immunohistochemical (IHC) findings of primary carcinoid tumor arising within mature teratoma of the kidney. (A) Cytokeratin 7 staining was positive in the urothelial-like epithelium, but negative in the carcinoid tumor cells and the colonic-like epithelium. Original magnification ×200. (B) Cytokeratin 20 staining was positive in the colonic-like epithelium, but negative in the carcinoid tumor cells and the urothelial-like epithelium. Original magnification ×400. (C) Synaptophysin staining was positive in the carcinoid tumor cells, but negative in the colonic-like epithelium and the urothelial-like epithelium. Original magnification ×200. (D) CD 56 staining was positive in the carcinoid tumor cells, but negative in the colonic-like epithelium and the urothelial-like epithelium. Original magnification ×200. (E) Both the colonic-like and urothelial-like epithelial lining of the teratomatous cystic spaces, and the carcinoid tumor cells showed positive pancytokeratin staining. Original magnification ×200. (F) Thyroxine staining was positive in the carcinoid tumor cells, but negative in the colonic-like epithelium and the urothelial-like epithelium. Original magnification ×200.

Immunohistochemical staining of the carcinoid tumor was performed in 6 of the 7 cases, but the panel used varied from case to case. Pancytokeratin was positive in all the carcinoid tumors in which it was used (Figure 3E) (100%; 4/4). The other immunohistochemical markers that were positive in all the carcinoid tumors in which they were used were Synaptophysin (Figure 3C) (100%; 3/3), Neuron specific enolase (100%; 3/3), Serotonin (100%; 2/2), CD 56 (Figure 3D) (100%; 1/1), Thyroxine (Figure 3F) (100%; 1/1), Glucagon (100%; 1/1), and Somatostatin (100%; 1/1). Chromogranin was positive in 4 out of the 5 cases (80%) in which it was used. S-100 protein was positive in 1 out of the 2 cases (50%) in which it was used. Immunohistochemical markers that were negative in all the carcinoid tumors in which they was used were Insulin (0%; 0/2), Cytokeratin 7 (Figure 3A) (0%; 0/1), Cytokeratin 20 (Figure 3B) (0%; 0/1), Calretinin (0%; 0/1), Calcitonin (0%; 0/1), Progesterone (0%; 0/1), Estrogen (0%; 1/1), Melan-A (0%; 1/1), Thyroid transcription factor-1 (0%; 0/1), Prostate specific antigen (0%; 0/1), Adrenocorticotropic hormone (0%; 0/1), Gastrin (0%; 0/1), Motilin (0%; 0/1), and Neurotensin (0%; 0/1).

The teratomatous cysts in our unpublished case were lined by urothelial-type epithelium (positive for Pancytokeratin and Cytokeratin 7, Figures 3A &3E) and focal colonic mucosa (positive for Cytokeratin 20, Figures 3B), but negative for Calretinin (a mesothelial marker, not shown).

## Treatment, follow-up and prognosis

Treatment and follow-up for the 7 cases of primary carcinoid tumor arising within mature teratoma of the kidney are summarized in Table 3[1-6]. Surgery was the only treatment in all cases. Of the 7 cases, 5 patients had nephrectomy and 2 had partial nephrectomy, and margins were negative in all 7 cases. No additional treatment was given in any of the 7 cases. Local recurrences and metastases were absent in all 7 cases. Follow-up was available for 3 of the 7 cases. All the 3 patients with complete follow-up were alive and well as of the publication of their respective cases (mean follow-up of 5 months). Serum chromogranin and urinary 5-HIAA levels were within normal limits in our unpublished case. Additionally, post-surgery octreotide scintigraphy performed in our unpublished case was negative, hence confirming the carcinoid tumor as originating primarily in the kidney (rather than elsewhere with metastasis to the kidney) and the absence of metastases from the renal carcinoid tumor.

**Table 3:** Treatment, follow-up and prognosis for primary carcinoid tumor arising within mature teratoma of the kidney

**Source, year**	**Primary Treatment**	**Final Margins**	**Additional Treatment**	**Pattern of Spread and Progression**		Outcome*
					
				**Local Recurrences**	**Metastases**	
Kojiro et al, 1976 [2]	Nephrectomy	Negative	No	No	No	NP
Fetissof et al, 1984 [3]	Nephrectomy	Negative	No	No	No	NP
Lodding et al, 1997 [1]	Nephrectomy	Negative	No	No	No	NP
Yoo et al, 2002 [4]	Nephrectomy	Negative	No	No	No	Alive and well with no evidence of disease 3 months after diagnosis
Kim et al, 2004 [5]	Nephrectomy	Negative	No	No	No	Alive and well with no evidence of disease 6 months after diagnosis
Kurzer et al, 2005 [6]	Partial Nephrectomy	Negative	No	No	No	NP
Current case	Partial Nephrectomy	Negative	No	No	No	Alive and well with no evidence of disease 6 months after diagnosis

*NP, not provided

## Discussion

Carcinoid tumors are characteristically low grade malignant tumors with neuroendocrine differentiation that have been described in several locations, including the lung, gastrointestinal tract, liver, kidney, ovary, bladder, testes and prostate [9]. Carcinoid tumors occur mostly in the gastrointestinal tract (74%) and bronchial system of the lungs (25%). In less than 1% of cases these tumors have been reported in the genitourinary system [9]. Carcinoid tumors of the kidney are among the most unusual of all renal neoplasms. Carcinoid tumors are thought to arise from enterochromaffin cells or amine precursor uptake and decarboxylation (APUD) cells, and are widely distributed throughout the body. In the urogenital tract, APUD cells have been described in the urinary bladder (especially in the region of the trigone), the prostate, the urethra, and the renal pelvis, but not in the renal parenchyma [9-12].

Primary carcinoid tumors of the kidney are rare, and primary carcinoid tumor arising within mature teratoma of the kidney is even rarer. Carcinoid tumors arising in urologic organs are rare, but have been reported in the kidney, prostate, and testes [7,9]. Only about 60 cases of carcinoid tumor of the kidney have been reported in the international medical literature [7,8], including 6 cases found as components of mature teratomas [1-6]. Although they exhibit morphologic and immunohistochemical features consistent with a hindgut neuroendocrine phenotype [13], the precise histogenesis of renal carcinoid tumors is uncertain and is a matter of speculation. The most popular hypothesis is that primary renal carcinoid tumor arise from multipotential primitive stem cells capable of neuroendocrine differentiation [7,12,14]. Although conclusive evidence for this theory is lacking at present, one renal carcinoid tumor has been shown to share some genetic aberrations with renal cell carcinomas, indicating a common genetic event in the tumorigenesis for these two entities [14].

Abundant published evidence support the notion that renal carcinoid tumors are derived from interspersed neuroendocrine cells associated with acquired and congenital renal abnormalities [3,4,7,8,12,13,15-18]. Cases of renal carcinoid tumor arising in association with renal teratoma and/or horseshoe kidneys lend weight to the theory that endocrine cells that comprise the carcinoid tumors may arise by divergent differentiation from a common stem cell, which also gives rise to the epithelial and mesenchymal components of the teratoma [3,4,18]. The relative risk of renal carcinoids in patients with horseshoe kidneys has been calculated at between 62% and 82% [13,16]. Five of the 7 cases of primary carcinoid tumor arising within mature teratoma of the kidney in this series had no relationship with a horseshoe kidney. Horseshoe kidneys have been proposed to be the result of teratogenic factors, which may also account for the increased risk of malignant tumors in horseshoe kidneys [1,13,16]. The increased incidence of carcinoid tumors in horseshoe kidneys is likely due to predisposing embryological factors or teratogenic events involving the abnormal migration of posterior nephrogenic cells, which coalesce to form the isthmus of horseshoe kidneys [16]. The primary location of all horseshoe kidney derived carcinoid tumors in the vicinity of the isthmus strongly supports this theory [16]. Carcinoid tumors occurring in renal teratomas are thought to be derived from neuroendocrine cells of the gastrointestinal and respiratory epithelium, which are components of these teratomatous lesions [4].

With the inclusion of our current unpublished case, 7 cases of primary carcinoid tumor arising within mature teratoma of the kidney formed the basis of our analysis and subsequent comments. Epidemiologically, primary carcinoid tumor arising within mature teratoma of the kidney occurred predominantly in the fourth to seventh decades of life, except one case occurring at age 23. The 7 reviewed cases showed no sex predilection, but the 2 cases of primary carcinoid tumor arising within mature teratoma in horseshoe kidneys occurred exclusively in men, likely reflecting the higher prevalence of horseshoe kidneys in male patients [19]. Surgical resection of these tumors accompanied by lymph node dissection was the primary and only treatment employed to achieve oncological cure in all the 7 cases herein reviewed.

The most common clinical finding in the 7 reviewed cases was abdominal or flank pain. However, about 28.6% of patients with primary carcinoid tumors arising within mature teratoma of the kidney were asymptomatic at the time of discovery, most probably due to their slow growth and hence often remaining clinically silent for many years before manifesting symptomatically. Clinically apparent carcinoid syndrome was absent in all the 7 reviewed cases, likely reflecting their hindgut origin [13] and the breakdown of their secreted biologically active hormones in the liver before reaching the systemic arterial circulation. The most common (71.4%) radiological feature of in the 7 reviewed cases was calcification. Although the renal lesions may be heterogeneous on CT, the typical imaging characteristic was complex cystic Bosniak II-III lesion with focal calcification and minimal enhancement on contrast enhanced CT.

The predominant histological architectural pattern of primary carcinoid tumor arising within mature teratoma of the kidney was trabecular or ribbon-like arrangement admixed with solid nests with peripheral palisading, focal rosette-like areas and rare mitotic events. Cytologically, the nuclei were uniformly round with "salt-and-pepper" chromatin pattern. Although these histological features are typical of carcinoid tumors and may assist with diagnosis, they give the lesion a pseudopapillary appearance, which may lead to misdiagnosis [20]. In a recent review of renal carcinoid tumors, the histopathological diagnosis was initially mistaken in 14.5% of reported cases [7], probably because of limited awareness about this pathological condition and the absence of carcinoid syndrome clinically. Immunohistochemically, synaptophysin, chromogranin and neuron specific enolase were the most valuable markers for the diagnosis of primary carcinoid tumor arising within mature teratoma of the kidney.

The 7 reviewed cases were usually diagnosed as large masses (mean size of 4.4 cm) because of the slow growth pattern of renal carcinoid tumors in the retroperitoneum, which is a highly distensible potential space. Despite the absence of follow-up data for some of the cases, the biologic behavior and prognosis of primary carcinoid tumor arising within mature teratoma of the kidney appeared excellent. None of the 7 patients had local recurrences or metastases, though follow-up was incomplete for 4 cases. The remaining 3 patients with complete follow-up were alive and well at the time of their respective publications (mean follow-up of 5 months). Hence, although definitive conclusions cannot be drawn from such a small set of patients without long term 5-year follow-up, the ultimate biologic behavior of primary carcinoid tumor arising within mature teratoma of the kidney may be excellent, and may be much better than that for carcinoid tumors elsewhere [9]. Interestingly, 1 previous report indicated that carcinoid tumors associated with horseshoe kidneys were apparently associated with a more benign course [16]. However, a recent review of renal carcinoid tumors revealed that neither renal teratoma nor horseshoe kidneys derived carcinoid tumors were associated with a better prognosis than carcinoid tumors originating in normal kidneys [7]. Furthermore, this recent review reported the presence of metastases in 45.6% of patients at initial diagnosis [7].

## Conclusion

Primary carcinoid tumor arising within mature teratoma of the kidney is a rare tumor that typically presents with abdominal or flank pain without carcinoid syndrome. It was associated with horseshoe kidneys, and hence the presence of horseshoe kidney on radiographic evaluation should lead to further careful investigation. Additionally, when a renal carcinoid tumor arising in mature teratoma is diagnosed, a search should be performed for a possible primary elsewhere. Although follow-up was lacking in some of the cases herein reviewed, primary carcinoid tumor arising within mature teratoma of the kidney was not associated with local recurrence and metastasis, was surgically curable, and had a rather favorable prognosis.

## Competing interests

The author(s) declare that they have no competing interests.

## Authors' contributions

**HBA **participated in the histopathological evaluation, performed the literature review, acquired photomicrographs and drafted the manuscript. **AVP **conceived and designed the study, gave the final histopathological diagnosis and revised the manuscript for important intellectual content. Both authors read and approved the final manuscript.
